# Antiretroviral therapy and population mortality: Leveraging routine national data to advance policy

**DOI:** 10.1371/journal.pmed.1002469

**Published:** 2017-12-12

**Authors:** Amitabh B. Suthar, Till Bärnighausen

**Affiliations:** 1 Centers for Disease Control and Prevention, Atlanta, Georgia, United States of America; 2 Africa Health Research Institute, Mtubatuba, South Africa; 3 Harvard T.H. Chan School of Public Health, Boston, Massachusetts, United States of America; 4 Heidelberg Institute of Public Health, Heidelberg, Germany

## Abstract

In a Perspective, Amitabh Suthar and Till Bärnighausen discuss progress made so far in reducing HIV-related mortality in South Africa and keys towards further population mortality reductions going forward.

## Antiretroviral therapy and population mortality

**Fig 1:**
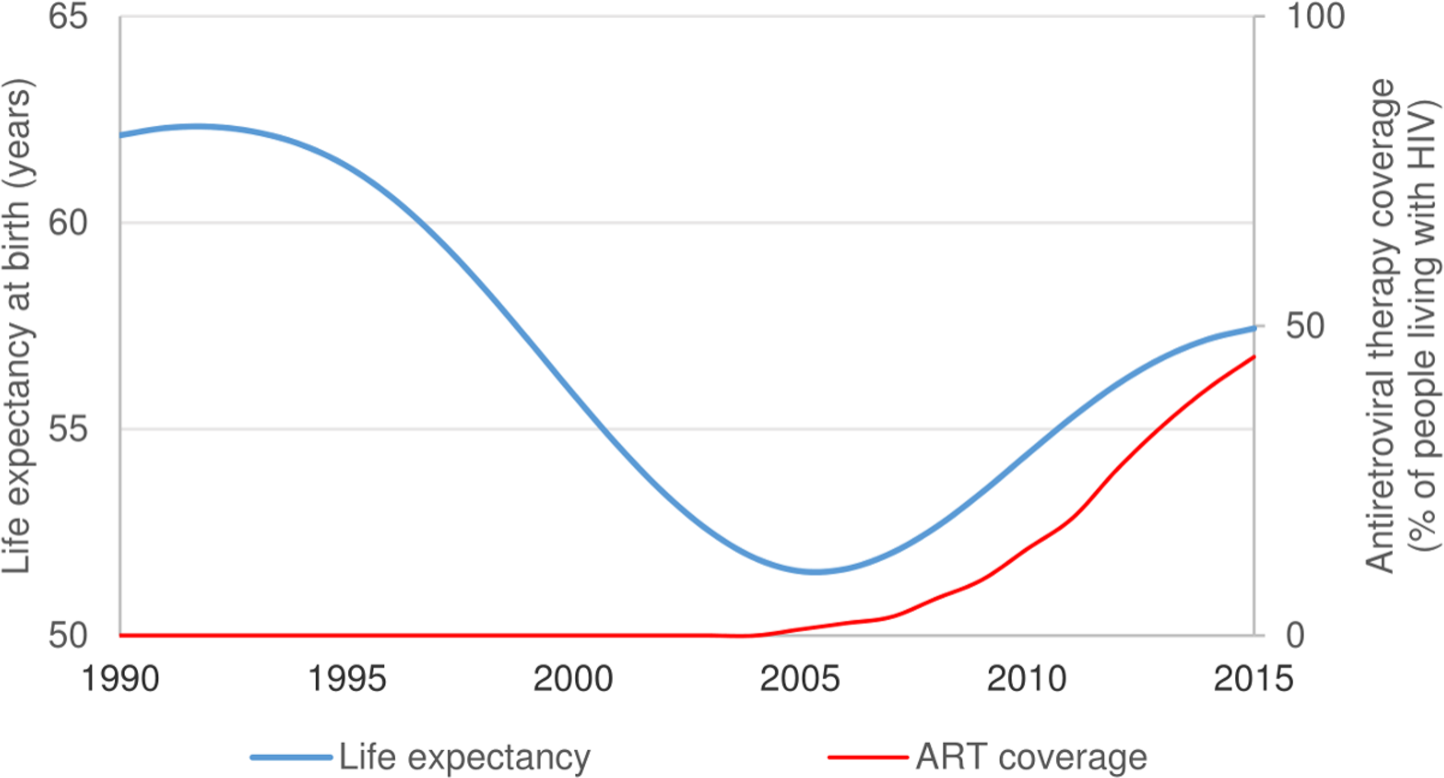
South African life expectancy and antiretroviral therapy (ART) coverage, 1990–2015 [2].

Reducing mortality from HIV, tuberculosis, and malaria with effective medicines, preventing diseases with immunisations, and improving access to safe water and sanitation are some of the greatest public health achievements of recent times [1]. According to World Bank estimates, the HIV epidemic coincided with reductions in life expectancy in South Africa that were largely reversed during the scale-up of antiretroviral therapy (ART) (Fig 1) [2]. In KwaZulu-Natal, the South African province with the most severe HIV epidemic, empirical data from one of Africa’s largest population cohorts has shown rapid life expectancy increases following South Africa’s national ART rollout [3].

This week in *PLOS Medicine*, Leigh Johnson and colleagues have now substantially advanced the evidence on the impact of mass ART on population mortality through a rigorous combination of empirical, national-level data on HIV prevalence and mortality [4]. They find that from 2000–2014 South Africa’s ART programme saved 1.72 million adult lives, adding 6.15 million years of life to the South African population. Johnson and colleagues also show that politically motivated delays in the provision of ART to South Africa’s population contributed to loss of an estimated 2.65 million life years, underscoring the importance of making prompt HIV treatment available to all. The estimated impact of the South African national ART programme is impressive and provides hope that HIV-related mortality could be virtually eliminated over the coming decades.

## Deaths across stages of the HIV treatment cascade

The HIV treatment cascade is considered a way to describe and analyse patient behaviour in the interval between HIV diagnosis and long-term retention on ART [5]. In 2014, Johnson and colleagues used a simulation model for South Africa to estimate that 18% of adult HIV-related deaths occurred in individuals who were undiagnosed, 41% occurred in individuals who were diagnosed and ART naïve, 10% occurred in individuals within six months of starting ART, and 30% occurred in individuals who had started ART more than six months prior to death [4]. These percentages are similar to empirical estimates using population-based data from KwaZulu-Natal, which showed that 55% of HIV-related deaths in men and 40% of HIV-related deaths in women occurred in individuals who were either undiagnosed or diagnosed and ART naïve [6]. The large losses of life that occur before ART initiation indicate a need for innovations to reach those who are not yet diagnosed with HIV or have not yet accessed HIV treatment services. Several recent innovations in HIV care could substantially reduce the mortality burden due to late diagnosis or poor HIV treatment access. First, HIV self-tests recently became available in pharmacies in South Africa [7]. Increased access to HIV self-testing—for instance, through community health worker distribution—could substantially improve knowledge of HIV status [8]. Second, not only did South Africa recently change its national ART guidelines to initiate ART in all people with HIV irrespective of their CD4 count, but the World Health Organization now recommends starting ART on the same day of diagnosis [9,10]. These changes are likely to reduce mortality amongst those patients who, up to now, would access HIV treatment services but would not make it through the multiple visits required to initiate ART. Third, future innovations in ART delivery approaches—such as community health worker-based ART delivery and vending machines that dispense antiretrovirals—are likely to improve access to ART and reduce mortality prior to ART initiation [11–13]. Updating estimates on the distribution of deaths across the HIV treatment cascade may support monitoring of the impact of these health systems innovations. More granular data with further sex, age, and geographic disaggregation may help identify additional service delivery challenges.

## Health systems innovations to reduce population mortality

Universally diagnosing people early in the course of HIV disease and immediately initiating ART is the most effective way to ensure that all theoretically preventable deaths from HIV are prevented. Recent guidelines to treat all people with HIV irrespective of their CD4 count should facilitate immediate ART initiation [10]. The national scale-up of ART has improved the national health service infrastructure to reduce HIV-related deaths. Looking forward, South Africa could achieve further population mortality reductions by leveraging this infrastructure to reduce non-HIV related deaths—such as those owing to stroke, hypertension, and diabetes—amongst people with HIV and the general population. New South African policy guidance to decongest HIV facilities by moving the treatment of stable ART patients into communities, as well as to reengineer national health insurance and primary care facilities to provide care for other chronic conditions, lays out a powerful vision of a health system that could achieve large mortality reductions over the coming decades [10,14].

## The value of routine national data

As Johnson and colleagues point out, one of the major methodological advances of their work is a mathematical model of the HIV epidemic that is calibrated not only to nationally representative HIV prevalence data but also to nationally representative mortality data [4]. As they indicate, relying on selected cohort data can lead to overrepresentation of urban facilities, underrepresentation of public health sector facilities, overrepresentation of high socioeconomic-status patients, and overrepresentation of facilities with high health staff-to-patient ratios [4]. By using national mortality data from the South African vital registration system, Johnson and colleagues avoid these biases, illustrating powerfully the value of mortality data that represent the nation rather than selected patient populations [4]. Although there remain challenges in relation to timeliness of registration, the coverage of South African death registration data has increased over the past two decades and has reached levels of above 90% population coverage in recent years, indicating that continuous and robust vital registration systems are possible in sub-Saharan Africa [15,16]. Additional national data could in the future expand the scope of simulation studies to capture the effects of alternative health systems delivery models on the impact of ART and other treatments. In South Africa, examples of such data include the health systems and clinical laboratory data available in the South African TIER.Net electronic patient management system and the National Health Laboratory Service [17]. Studies using these data could generate novel insights into approaches to reduce population mortality through health systems innovation.

## Promoting good policy in the Sustainable Development Goals era

During the era of the Millennium Development Goals, ART was one of the main instruments available to achieve one of only eight global development goals [1]. The Sustainable Development Goals broadened the overall development agenda and the focus on health within it. As a result, in South Africa and elsewhere, Ministers of Finance are facing increasingly difficult decisions in prioritising interventions to achieve ambitious domestic and global development agendas across sectors [18]. At the same time, the global commitments to fight the HIV epidemic are weakening while the number of people needing ART is increasing [19]. While impact data, such as those generated by Johnson and colleagues, are powerful, they may be insufficient on their own to influence policy and resource allocation. The decisions required for progressing today’s development agenda with governments and nonstate actors (e.g., companies, high net-worth individuals, and philanthropic foundations) require metrics that compare interventions across sectors, such as return on investment.

Cost–benefit analyses that span interventions across sectors, such as those commissioned by the Copenhagen Consensus Center, are a good starting point for multisectoral prioritisation for development [20]. In these analyses, ART is compared with other interventions in the HIV sector (such as male medical circumcision), in the larger health sector (such as contraception and interventions for tuberculosis and child malnutrition), and in all other sectors (such as increasing preschool access, reducing tax evasion, and trade liberalisation) [21]. However, multisectoral competition for resources should rest not only on the standard cost–benefit criterion but also on other criteria [22], such as the magnitude of the problem an intervention addresses, the system capacity to deliver the intervention, and the effects of the intervention on equity and human rights [23]. For example, some have argued that trade liberalisation enhances economic growth for the entire population of a country, while investments in tuberculosis and child malnutrition predominantly improve health amongst poor and vulnerable groups [24–26]. One other criterion in multisectoral decision-making should be the strength of the intervention’s evidence of impact used in cost–benefit analyses. All else being equal, we should prefer to invest in an intervention whose anticipated impact is more certain over one whose impact is uncertain [27]. The study by Johnson and colleagues substantially strengthens the evidence on the population health impact of scaling up ART in South Africa [4]. It is in this regard that we hope it will be influential for South African and global health policy.

## Abbreviations

ART: antiretroviral therapy
